# Comparison of acute outcomes from elective total hip replacements and after fragility femoral neck fractures in nonagenarians

**DOI:** 10.1186/s12891-024-07340-1

**Published:** 2024-04-24

**Authors:** Zahra Al-Essah, Keegan Curlewis, Gareth Chan, Karim Tokeisham, Koushik Ghosh, Philip Stott, Benedict A. Rogers

**Affiliations:** 1 Brighton and Sussex Medical School, Brighton, UK; 2 Royal Free NHS Foundation Trust, London, UK; 3 University Hospitals NHS Foundation Trust, Sussex, UK

**Keywords:** Total hip replacement, Neck of femur, Femoral neck fracture, Nonagenarians

## Abstract

**Background:**

Hip hemiarthroplasty has traditionally been used to treat displaced femoral neck fractures in older, frailer patients whilst total hip replacements (THR) have been reserved for younger and fitter patients. However, not all elderly patients are frail, and some may be able to tolerate and benefit from an acute THR. Nonagenarians are a particularly heterogenous subpopulation of the elderly, with varying degrees of independence. Since THRs are performed electively as a routine treatment for osteoarthritis in the elderly, its safety is well established in the older patient. The aim of this study was to compare the safety of emergency THR to elective THR in nonagenarians.

**Methods:**

A retrospective 10-year cohort study was conducted using data submitted to the National Hip Fracture Database (NHFD) across three hospitals in one large NHS Trust. Data was collected from 126 nonagenarians who underwent THRs between 1st January 2010 – 31st December 2020 and was categorised into emergency THR and elective THR groups. Mortality rates were compared between the two groups. Secondary outcomes were also compared including postoperative complications (dislocations, revision surgeries, and periprosthetic fracture), length of stay in hospital, and discharge destination.

**Results:**

There was no significant difference in mortality between the two groups, with 1-year mortality rates of 11.4% and 12.1% reported for emergency and elective patients respectively (*p* = 0.848). There were no significant differences in postoperative complication rate and discharge destination. Patients who had emergency THR spent 5.56 days longer in hospital compared to elective patients (*p* = 0.015).

**Conclusion:**

There is no increased risk of 1-year mortality in emergency THR compared to elective THR, in a nonagenarian population. Therefore, nonagenarians presenting with a hip fracture who would have been considered for a THR if presenting on an elective basis should not be precluded from an emergency THR on safety grounds.

**Trial registration:**

Not necessary as this was deemed not to be clinical research, and was considered to be a service evaluation.

## Background

Femoral neck fractures (FNFs) are a global healthcare burden, and a leading cause of hospital admissions among the elderly in the United Kingdom (UK) with an associated 30-day mortality rate of 6.5% [1]. Currently, the UK National Health Service (NHS) treats 75,000 hip fractures annually, costing more than £2 billion per year [2]. As the UK’s population ages, hip fracture incidence is expected to rise, with costs projected to reach £3.5—£5.6 billion by 2033 [3].

Total hip replacement (THR) or hemiarthroplasty are the treatment of choice for displaced intracapsular hip fractures. Traditionally hemiarthroplasty is preferred for older, frailer patients as it is associated with fewer postoperative complications [4]. National Institute for Health and Clinical Excellence (NICE) recommends that a THR should be considered in patients who are independently mobile with no more than 1 stick, medically fit for anaesthesia and the procedure and expected to remain independent for more than 2-years post-surgery. There is the specific omission of any age barriers in the recommendation [1–3]. These guidelines are based on potential improved long-term functional outcomes in THR over a HA in fit and active patients [5, 6]. Despite NICE guidelines, there are still disparities in the use of THR nationally, with only a third of eligible candidates receiving THR in the UK [7]. These values vary considerably across England’s NHS Trusts, with the proportion of eligible patients undergoing THR ranging from 6 – 70% [1, 8]. This may be in part due to conflicting evidence in the support of THRs. [9–12].

The HEALTH study suggested equitable patient reported outcomes and operative complications at 2-years when comparing hemiarthroplasty and THR in intra-capsular fractures. However, the relative benefits of a THR may not have become apparent during this follow-up period and longer-term results are needed. It should be accepted that a THR is a more complex procedure that is associated with a higher risk of dislocation than hemiarthroplasty [13, 14].

Nonagenarians are subpopulation of the elderly aged between 90 to 99 years old, and are a very heterogeneous population; ranging from independent and fit to medically frail and dependent. Improvements in the management of chronic conditions and healthier lifestyle choices have led to the elderly living longer and becoming increasingly independent [15, 16]. Healthcare professionals are seeing a greater number of nonagenarians, and they are often described as a uniquely heterogenous population. While some nonagenarians are frail and medically comorbid, other individuals live into their 90 s in good health, with active lifestyles, and have few medical comorbidities [17]. *Lowksy* *et. al* [18]*.* demonstrated that this variation in health status in the elderly is influenced by numerous social factors including gender, race, income, and educational attainment, suggesting that poor health is not always a consequence of older age.

The healthier subpopulation of nonagenarians frequently meet the criteria for a THR but are often not offered one, potentially due to underlying clinical decision making biases. The decision for a hemiarthroplasty in frail nonagenarians is straightforward, as these patients have a high risk of postoperative complications [19], and need a quicker less invasive operation; a hemiarthroplasty. However, the choice is less clear in healthier nonagenarians. Previous studies have shown that patients who satisfy the NICE criteria have lower mortality with THR than hemiarthroplasty [5]. Conversely, those who do not satisfy the criteria have worse mortality [20].

This study primarily aims to assess the safety of nonagenarians who meet the criteria and underwent a THR after a NOF fracture as per NICE guidance.

### Aims

This study aims to assess whether THRs in unplanned cases due to femoral neck fractures in nonagenarians is of comparable safety when compared to planned THRs in nonagenarians.

## Methods

### Study design

A retrospective cohort analysis of a prospectively collected database of all THR performed between 1st January 2010 and 31st December 2020 at three NHS Acute Hospitals comprising three district general hospitals (Teaching Hospital with Level 3 trauma centre capabilities) and a regional Major Trauma Centre (Teaching Hospital with Level 1 trauma capabilities) was performed.

All three hospitals (Royal Sussex County Hospital, Brighton and Princess Royal Hospital, Haywards Heath; Worthing Hospital, Worthing; and St Richards Hospital, Chichester) have since merged to form a single NHS Trust. However, all hospitals still function as independent acute care providers.

### Patient selection

All consecutive patients undergoing a THR during the study period were identified through a prospectively collected local electronic database. Results were sequentially screened to identify only those patients aged 90 + years on the date of surgery and to identify those undergoing a THR for an FNF. These patients were defined as those patients whose radiographic records confirmed a FNF and whose care was eligible for payment by the NHS’ Best Practice Tariff (BPT) for fragility FNFs [21]. The BPT is a national payment by results mechanism which requires the submission of all fragility FNFs to the National Hip Fracture Database (NHFD). National ascertainment rates for fragility FNFs captured by the BPT and NHFD are in excess of 99% [22]. All other patients were assumed to be elective THR cases.

To improve the ascertainment of THR for trauma patients, the NHFD submissions for the study period were further interrogated to identify those treated with a hemiarthroplasty. Radiographic records were cross referenced to confirm the use of a hemiarthroplasty, those patients found to have been treated instead with a THR were then subsequently included for analysis as an emergency THR.

Patients undergoing a THR having previously been added to a waiting list for the procedure were categorised as an “elective THR”, whilst those who had a THR with immediate preceding radiological documentation of a FNF were classed as “emergency THR”. The elective THR cohort was considered to be the “control population” for this study in comparing the safety of unplanned emergency THRs in nonagenarians. Patients having been on the waiting list for an elective THR, but subsequently sustained a fragility FNF requiring a THR were analyses in the emergency THR cohort, as they did not have the routine pre-operative optimisation offered to our elective THRs. 

### Data collection

Patient demographics, indications for elective THR, discharge locations and postoperative outcomes were collected from the electronic patient and radiographic records. Indications for surgery, surgical approach, preoperative Abbreviated Mental Test Score (AMTS) and American Society of Anaesthesiology (ASA) grade were determined from electronic operative records. Radiographic records were assessed for radiological evidence of dislocations, periprosthetic fractures and revision prostheses implanted after the index THR.

The Nottingham Hip Fracture Score (NHFS): Preoperative AMTS, Preoperative haemoglobin (Hb) levels, Discharge Destination, Comorbidities, and History of malignancy [21]. This score was used to predict a patient’s 30-day mortality as part of our standardised consenting process. Charlson comorbidity index (CCI) was calculated from the collected data for all patients.

### Inclusion and exclusion criteria

All patients undergoing a primary THR aged between 90–99 years on the date of surgery were eligible for inclusion. Patients who received a THR as a revision procedure for a primary THR already in situ were excluded.

### Standards of care

All study hospitals 7-days a week, 365-day Consultant (Attending) Trauma & Orthopaedic delivered trauma surgery care, with access to dedicated trauma theatre lists with appropriate pre- and peri-operative Consultant Anaesthesiologist care with pre-operative optimisation and post-operative rehabilitation provided by Orthogeriatric Physicians.

Post-operative regimes naturally had some variance between hospitals and surgeons, but however several consistent themes were adopted across the board; (a) immediate weight-bearing in the post-operative period, (b) day 1 post-operative physiotherapy rehabilitation and (c) venous thromboprophylaxis in accordance with the NICE guidance at the time of surgery [23].

### Study endpoints

The primary outcome of this study was to determine the safety of emergency THR performed in nonagenarians compared to elective THR performed in nonagenarians by comparison of mortality rates (30-day and 1-year mortality).

The secondary outcomes of this study were to compare the postoperative complication rates of nonagenarians undergoing emergency THR to nonagenarians undergoing elective THR including: rates of dislocation, rates of periprosthetic fracture and rates of revision surgery, as well as the length of stay in hospital and discharge destinations following surgery in nonagenarians undergoing emergency THR to nonagenarians undergoing elective THR.

### Ethical approval

The Medical Research Council and National Health Service (NHS) Health Research Authority decision tool was completed and the study was considered “not to be researched by the NHS”. As such local ethical approval was not required for this service evaluation study [24].

### Statistical analysis

Statistical analysis was conducted using IBM SPSS 27.0 Software for Macintosh (SPSS, Inc., Chicago, IL, USA). Univariate analyses were conducted to compare data between two independent study groups. The Shapiro–Wilk test was used to assess normality of distribution for continuous data. Variables that deviated from normality were analysed using the two-sided Mann–Whitney U test whereas normally distributed data was analysed using the independent samples t-test. Categorical data was analysed with Chi-squared test (χ^2^ test) or Fisher’s exact test. Cumulative mortality was estimated using Kaplan–Meier analysis.

Numerical data was reported as medians with interquartile ranges (IQR) as well as mean ± standard deviation (SD). Categorical data was expressed as frequencies and percentages.

## Results

**Fig. 1:**
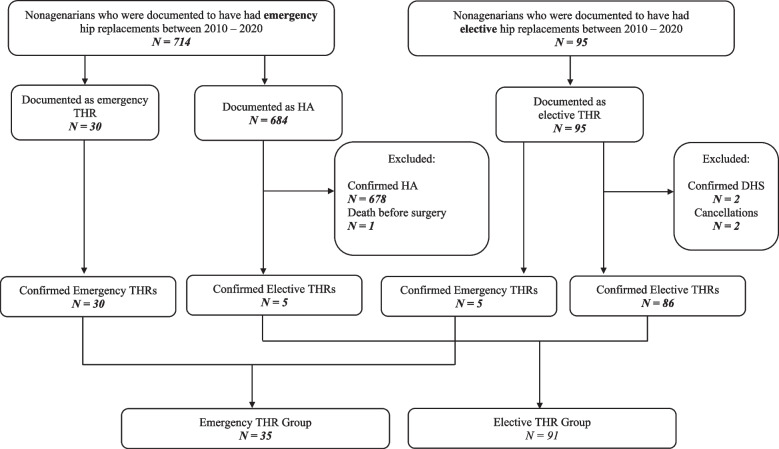
Study flow diagram showing inclusion and exclusion criteria of patients Abbreviations: THR – Total Hip Replacement; HA – Hemiarthroplasty; DHS – Dynamic Hip Screw

A total of 126 patients were eligible for inclusion having received a THR on either an elective basis or for a traumatic FNF (Fig. 1).

### Indications for surgery

Out of a total of 126 nonagenarians who underwent a THR between 2010 – 2020, 35 patients received emergency THR for FNF fractures and 91 patients had elective THR, with the commonest reason being Osteoarthritis (60/91) (Tables 1 and 2).

**Table 1:** Indications for surgery of nonagenarians who had emergency total hip replacements between 2010 – 2020

Indications for surgery	Emergency THR Group *N* = *35*
Intertrochanteric NOF fracture (n)	4
Intracapsular NOF fracture (n)	24
Other (n)	7

**Table 2:** Indications for surgery of nonagenarians who had elective total hip replacements between 2010 – 2020

Indications for surgery	Elective THR Group *N* = *91*
Osteoarthritis (n)	60
Osteoarthritis secondary to AVN (n)	3
Revision of previous hip fracture procedure (n)	8
Delayed hip fracture (n)	7
Cancer metastasis to hip (n)	1
Unknown	1

### Patient characteristics

Preoperative patient characteristics are displayed in Table 3. Mean patient age was 91.8 years in the emergency group (range 90–99 years). Mean age in the elective group was 92.0 years (range 90–98 years). Females made up 65.7% (*n* = 23) of the emergency group, and 79.1% (*n* = 72) of the elective group (*p* = 0.118). Overall, the two surgical groups were comparable and equivalent; there were no statistically significant differences in baseline characteristics (*p* > 0.05).

**Table 3:** Comparison of preoperative baseline patient characteristics of patients who underwent emergency total hip replacement or elective total hip replacement

**Variables**		**Emergency THR Group**^**a**^*N* = *35*	**Elective THR Group**^**a**^*N* = *91*	***p-value***^***2***^
**Age** (years)	Mean ± SD	91.82 ± 2.289	92.01 ± 1.964	*0.651*
	Median (IQR)	91 (90 – 93)	91 (90 – 93)	
**Sex** [n (%)]	Male	12 (34.3)	19 (20.9)	*0.118*
	Female	23 (65.7)	72 (79.1)	
**AMTS** [n (%)]	< 7	1 (2.9)	3 (3.3)	*0.316*
	> 7	16 (45.7)	15 (16.5)	
	Missing data	18 (51.4)	73 (80.2)	
**ASA** [n (%)]	1	0 (0.0)	1 (1.1)	*0.440*
	2	13 (37.1)	25 (27.5)	
	3	11 (31.4)	41 (45.1)	
	4	3 (8.6)	5 (5.5)	
	Missing data	8 (22.9)	19 (20.9)	
**Presence of comorbidities**	Yes	21 (60.0)	67 (73.6)	*0.136*
	No	14 (40.0)	24 (26.4)	
**CCI** [n (%)]	4	16 (45.7)	33 (36.3)	*0.665*
	5	5 (14.3)	20 (22.0)	
	6	6 (17.1)	12 (13.2)	
	7	3 (8.6)	11 (12.1)	
	8	0 (0.0)	3 (3.3)	
	9	0 (0.0)	2 (2.2)	
	10	0 (0.0)	1 (1.1)	
	Missing data	5 (14.3)	9 (9.9)	
**Smoking status**	Current smokers	0 (0.0)	4 (4.4)	0.017*
	Ex-smokers	3 (8.6)	14 (15.4)	
	Non-smokers	12 (34.3)	10 (11.0)	
	Missing data	20 (57.1)	63 (69.2)	
**Preoperative Hb** (g/L)		120.86 ± 13.746	112.40 ± 30.010	*0.155*

*Abbreviations*: *THR *Total Hip Replacement, *AMTS *Abbreviated Mental Test Score, *ASA *American Society of Anaesthesiology Score, *CCI *Charlson Comorbidity Index, *Hb *Haemoglobin, *n *number

^a^Mean ± standard deviation, or median (IQR) or frequency (percentage)

^2^ – Mann Whitney U test, Chi-squared test, or Independent Samples T test

### Operative data

Operative data including side of fracture, type of implant and surgical approach are represented in Table 4.

**Table 4:** Operative data on patients who underwent emergency total hip replacement and elective total hip replacements

		**Emergency THR Group**^**a**^*N* = *35*	**Elective THR Group**^**a**^*N* = *91*	***p-value***^***2***^
**Side** [n (%)]	Left	20 (57.1)	41 (45.1)	*0.224*
	Right	15 (42.9)	50 (54.9)	
**Type of implant** [n (%)]	Hybrid	7 (20.0)	29 (31.9)	*0.224*
	Cemented	25 (71.4)	49 (53.8)	
	Uncemented	1 (2.9)	2 (2.2)	
	Missing data	2 (5.7)	11 (12.1)	
**Approach** [n (%)]	Posterior	22 (62.9)	47 (51.6)	*0.321*
	Anterolateral	5 (14.3)	20 (22.0)	
	Lateral	1 (2.9)	7 (7.7)	
	Missing data	7 (20.0)	17 (18.7)	

* Abbreviations*: *THR *Total Hip Replacement, *n *number

^a^Frequency (percentage)

^b^Fisher’s exact test or Mann Whitney U test

^*^ – Significant *p*-value

### Baseline comorbidities

A total of 17 patients in the emergency group and 60 patients in the elective group had medical comorbidities (Table 5). On the other hand, 31 patients did not have any background comorbidities, 11 of whom were in the emergency group and 20 in the elective group. Baseline comorbidities were not statistically different between the two surgical groups, (*p* > 0.05).

**Table 5:** Comparison of baseline comorbidities of patients who underwent emergency total hip replacements and elective total hip replacements

**Comorbidity**	**Emergency THR group**^**a**^*N* = 35	**Elective THR group**^**a**^*N* = 91	***p-value ***^***2***^
**Hypertension** [n (%)]	18 (51.4)	54 (59.3)	*0.836*
**Myocardial infarction** [n (%)]	0 (0.0)	9 (9.9)	*0.110*
**Congestive heart failure** [n (%)]	4 (11.4)	5 (5.5)	*0.237*
**Stroke or TIA** [n (%)]	6 (17.1)	7 (7.7)	*0.098*
**Dementia** [n (%)]	1 (2.9)	7 (7.7)	*0.679*
**COPD** [n (%)]	1 (2.9)	8 (8.8)	*0.444*
**Connective tissue disease** [n (%)]	1 (2.9)	7 (7.7)	*0.679*
**Liver disease** [n (%)]	0 (0.0)	1 (1.1)	*1.000*
**Chronic kidney disease** [n (%)]	4 (11.4)	20 (22.0)	*0.307*
**Diabetes** [n (%)]	1 (2.9)	10 (11.0)	*0.285*
**Active malignancy** [n (%)]	2 (5.7)	10 (11.0)	*0.730*
**Malignancy in the last 20 years** [n (%)]	3 (8.6)	16 (17.6)	*0.396*

*Abbreviations: THR *Total Hip Replacement, *TIA *Transient Ischaemic Attack*, COPD *Chronic Obstructive Pulmonary Disorder *n *number

^a^Frequency (percentage)

^2^Fisher’s exact test

### Mortality rates

There was no statistically significant difference in the mortality rates between both groups, including 30-day and 1-year mortality (Table 6). One patient from each group died within 30 days of surgery. A total of 15 patients died within a year of surgery, 4 of whom were in the emergency THR group and 10 in the elective THR group.

**Table 6:** Comparison of postoperative outcomes of patients who had emergency total hip replacements and elective total hip replacements *Abbreviations: THR – Total Hip Replacement; n – number; %* ^*a*^ *– percentage of the total deceased population; %* ^*b*^ *– percentage of the total study population*

Outcomes		Emergency THR Group^a^*N* = 35	Elective THR Group^a^*N* = 91	*p-value*^*2*^
Survival status [n(%^a^)]	Total Deceased	23	51	0.323
	Died within 30 days	1 (2.9)	1 (1.1)	0.568
	Died between 30 days and 1 year	4 (11.4)	11 (12.1)	0.848
Discharge destination [n(%^b^)]	Temporary place of residence	13 (37.1)	26 (28.6)	0.077
	Usual place of residence	9 (25.7)	43 (47.3)	
	Death prior to discharge	0 (0.0)	1 (1.1)	
	Missing data	13 (37.1)	21 (23.1)	
Length of stay in hospital (days)	Mean ± SD	16.00 ± 9.653	10.44 ± 10.456	0.015*
	Median (IQR)	12 (8 – 18)	8 (5 – 12)	
Dislocations [n]		0	0	-
Revision Surgery [n]		1	0	0.070
Periprosthetic Fracture [n]		0	2	-

^a^Mean ± Standard deviation or Frequency (percentage)

^2^Chi-squared Test, or Mann Whitney U Test

^*^Significant *p*-value

Overall, 13 (48.1%) patients in the emergency group died during the study period, and 37 (46.3%) patients died from the elective group. Average time to death was 93.2 months and 37.4 months for emergency and elective groups respectively.

### Complication rates

There were no reported dislocations in either group. Periprosthetic fractures were reported in two patients who underwent elective THRs (Table 6). No periprosthetic fractures were reported in the emergency THR group. One patient in the emergency THR group received postoperative revision surgery of their acetabular component. No other revision surgeries were reported.

### Length of stay in hospital

**Fig. 2:**
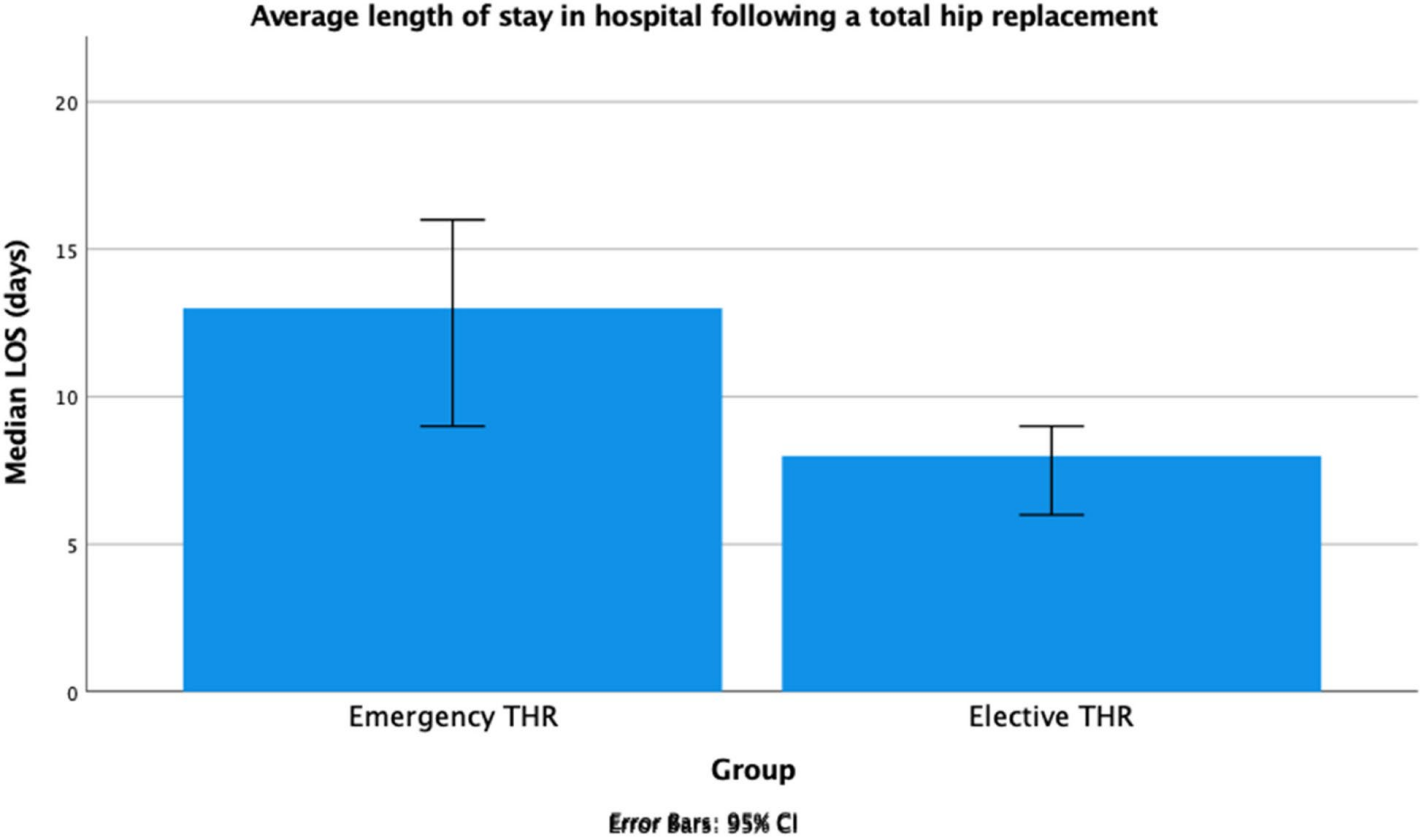
Median length of stay in hospital (days) of patients who received emergency THRs and patients who received elective THRs

Length of stay (LOS) in hospital was significantly longer in the emergency group compared to elective group (*p* = 0.015) (Table 6). Emergency patients spent on average 5.56 days longer in hospital compared to elective patients (Fig. 2).

### Discharge destination

Discharge destination was collected on 107 patients. There was no statistically significant difference in discharge location between the two cohorts (Table 6). In total, 7 patients from the emergency group and 15 from the elective group were discharged to a temporary place of residence. This includes NHS run- or private-care homes, rehabilitation centres or another NHS hospital. On the other hand, 9 emergency patients and 34 elective patients returned to their usual place of residence.

## Discussion

This study compared the surgical outcomes of emergency and elective THR, and found:

1. No significant difference in 30-day and 1-year mortality
2. No significant difference in postoperative outcomes, including rates of dislocation, periprosthetic fracture and revision surgery
3. Longer hospital stays in emergency patients

### Main finding

This study has found that THRs performed emergently for fragility FNFs are safe, with equitable outcomes compared to THR performed for elective indications in nonagenarians.

No significant difference in mortality rates following a THR in trauma or elective patients was identified at both 30-days and 1-year. This contrasts with previous studies, which demonstrated higher mortality rates in patients undergoing non-elective THRs compared to elective THRs [22, 25, 26]. However, the emergency cohorts in these studies were older, had more comorbidities, and were more frequently male, which are known risk factors for mortality. *Le Manach et. al* [25]*.* attempted to control for these by cofounding characteristics in their post-hoc model. However, many known risk factors of mortality were not included in the model, including ASA grade, preoperative Hb levels and baseline functional status [27–29]. *Xue et. al.*, with a similar patient cohort to this study matched their emergency and elective cohorts according to baseline characteristics, together with comorbidities, functional capacity, anaesthesia, and operative duration did not find significant differences in mortality [30].

The reported 1-year mortality rates of 11.4% for trauma patients shown in this study respectively are lower than the national United Kingdom average of 33.3% [31].

### Postoperative outcomes

There were only a small number of recorded complications in this study, with no dislocations, two periprosthetic fractures and one revision surgery reported overall. Our complication rates are substantially lower than previously reported rates [32]. This may be the result of a healthier cohort of nonagenarians in our study and/or the relative rarity of these complications and our comparatively small cohort size due to the unique nature of this patient demographic.

CCI scores did not surpass 10 for any patient, with most patients scoring 4 in both groups, solely due to their age. The reduced complication rate may also be explained by improvements in patient optimisation in elective patients, surgical technique, anaesthesia, wound care, and early mobilisation [33, 34].

The periprosthetic fractures both occurred in the elective group, while none were noted in the emergency group. This is inconsistent with past research which has shown that FNF patients have a higher risk of postoperative adverse outcomes compared to elective THR patients [35, 36]. This may reflect a healthier nonagenarian population in general and is likely to be reflective of appropriate patient selection prior to surgery but is likely to be due to the relatively small study size compared to the incidence rate. The emergency cohort had fewer comorbidities (CCI < 7) than the elective cohort, did not include any smokers, had higher AMTS scores and lower ASA grades, though these differences were not significant. This represents a selection bias where healthier nonagenarians are chosen for emergency THR, as per NICE guidance. The results of our study can be used to verify the safety profile of THR in selected nonagenarians, as many previous studies have done in the past [10, 11, 37].

### Patient selection for emergency THR

Age alone is not an adequate indicator of a patient’s eligibility for a THR, as evidenced by our findings and supported by NICE guidelines. Despite guidelines to this effect, only a third of eligible candidates receive THRs nationally due to the presumption that older patients are not fit for THRs [7]. Preoperative hip fracture scoring systems can be used as adjuncts with NICE criteria to reduce ambiguity in clinical decision-making. The Nottingham Hip Fracture Score (NHFS) is a validated tool to predict mortality after a Fragility FNF [38]. It risk stratifies based on the patient’s age, sex, AMTS, preoperative Hb, residence and comorbidities. A cut-off of 3 has been used to define premorbid status in Fragility FNF patients and can be used as a potential surrogate marker to define fitness for THR [39].

### Length of stay in hospital

Emergency patients in our study spent longer in hospital than elective patients, in accordance with previous studies [25, 40]. This disparity in LOS can be explained by the extensive preoperative planning and patient optimisation that goes into an elective procedure compared to an emergency admission [41]. Enhanced preoperative optimisation has consistently been shown to reduce LOS [42–44]. Differences in LOS of emergency and elective THRs may be explained by other confounding factors including characteristics such as sex, ASA grade, medical history and race [45, 46]. The HIP ATTACK study demonstrated a 1-day reduction in LOS but though no other benefits in mortality reduction in patients treated with an accelerated recovery programme relative to standard care in cases of FNFs [47]. *Schneider et*. *al.* [48] attributed FNF operative procedures themselves to increased LOS. However, only 3% of patients who spent longer than 14 days in hospital received a THR, with the rest (97%) receiving internal fixation or HA. Postoperative protocols that aim to minimise complications also substantially reduce LOS in hospital [49, 50]. Therefore, the true difference in the LOS resulting from whether a THR is an emergency or elective may be overstated. This also implies there are alternative targets to improve LOS for emergency patients such as preoperative patient selection and optimisation, and postoperative care.

### Reducing LOS in practice

Currently, fragility FNF patients occupy 1.5 million bed days per year in the NHS with an average LOS of 15.9 days [51]. Reducing LOS will enable beds to be more quickly available, allowing patients to receive more timely care whilst improving hospital efficiency, whilst reducing the risk of developing associated complications of increased LoS; e.g. healthcare acquired pneumonia, urinary tract infections and general deconditioning. Both preoperative and postoperative measures can be taken to reduce overall LOS for emergency THR patients.

Finally, delays in postoperative discharge have been regarded an important factor in a patient’s LOS in hospital. Despite the known benefits of rehabilitation on NOF patients, only 28.6% of U.K. NHS Hospitals discharge their patients to rehabilitation centres as soon as they become medically fit [52]. This is due to shortages in social care provisions, with patients not uncommonly waiting for care packages to become available thereby artificially extending their LOS for non-medical reasons.

### Limitations

The retrospective design of this study precludes its ability to draw causal conclusions. Secondly, the quality of the study’s findings was dependent on the quality of the data collected at the time. Unfortunately, because of variable record keeping, a proportion of patient data was unavailable; particularly preoperative AMTS scores. Selection bias may have been present in this study: selection of healthier nonagenarians for emergency THR may have resulted in the low mortality and comorbidity rates seen. Additionally, data was collected over a 10-year period, over which surgical protocols and incentives changed, including the implementation of the BPT in 2014, which may have influenced the results. The introduction of the BPT in the United Kingdom has been demonstrated to have improved the management and outcome of fragility FNF patients [53].

The study was also limited by the small sample size, reflective of the fact that nonagenarians are less frequently operated on in an emergency setting. A contributing reason for the small sample size was troubles with data ascertainment.

## Conclusion

This study showed that emergency THR is a safe procedure associated with similar incidence of mortality and postoperative outcomes as elective THR. It is safe for a carefully selected subset of nonagenarians who have sustained a displaced FNF to undergo a THR, and their age should not be a perceived contraindication to a THR. The authors believe that applying the test of whether the patient would have been offered an elective THR for degenerative causes, but subsequently presents with an acute fracture indicating the need for a THR is a reasonable and safe basis for clinical practice.

Hospital stay was longer in emergency patients, as expected with non-elective procedures. However, the predictors of hospital stay identified in this study may have clinical value. Patients undergoing THR should be screened preoperatively for risk factors of poor surgical outcome. Preoperative planning will allow for risk stratification as well as patient optimisation prior to surgery, ultimately improving patient outcomes and minimising hospital stay.

## Data Availability

Study data will not publicly available due to the inclusion of hospital performance data. Reasonable requests for the dataset will be facilitated where possible through communication with the corresponding author.
